# Situs inversus and ciliary abnormalities: 20 years later, what is the connection?

**DOI:** 10.1186/s13630-014-0010-9

**Published:** 2015-01-14

**Authors:** Petra Pennekamp, Tabea Menchen, Bernd Dworniczak, Hiroshi Hamada

**Affiliations:** Department of General Pediatrics, University Children’s Hospital Muenster, 48149 Muenster, Germany; Department of Human Genetics, University Hospital Muenster, 48149 Muenster, Germany; Graduate School of Frontier Biosciences, Osaka University, Osaka, Japan

**Keywords:** *Situs inversus*, Cilia, Left-right organizer, Node, *Nodal*, Nodal flow, Two cilia model, Planar cell polarity, Motile cilia, Sensory cilia

## Abstract

Heterotaxy (also known as *situs ambiguous*) and *situs inversus totalis* describe disorders of laterality in which internal organs do not display their typical pattern of asymmetry. First described around 1600 by Girolamo Fabrizio, numerous case reports about laterality disorders in humans were published without any idea about the underlying cause. Then, in 1976, immotile cilia were described as the cause of a human syndrome that was previously clinically described, both in 1904 by AK Siewert and in 1933 by Manes Kartagener, as an association of *situs inversus* with chronic sinusitis and bronchiectasis, now commonly known as Kartagener’s syndrome. Despite intense research, the underlying defect of laterality disorders remained unclear. Nearly 20 years later in 1995, Björn Afzelius discussed five hypotheses to explain the connection between ciliary defects and loss of laterality control in a paper published in the *International Journal of Developmental Biology* asking: ‘Situs inversus and ciliary abnormalities: What is the connection?’. Here, nearly 20 research years later, we revisit some of the key findings that led to the current knowledge about the connection between *situs inversus* and ciliary abnormalities.

**Electronic supplementary material:**

The online version of this article (doi:10.1186/s13630-014-0010-9) contains supplementary material, which is available to authorized users.

## Review

### Laterality defects in humans

Left-right asymmetry is common in nature. Disorders of left-right asymmetry can cause randomization (heterotaxy/*situs ambiguous*) or complete reversal of organ position (*situs inversus totalis*) (reviewed in [1-3]). The first reports describing laterality defects came from Girolamo Fabrizio (Hieronymus Fabricius; 1537 to 1619; Italian anatomist and surgeon) around 1600, followed by Marco Aurelio Severino (1580 to 1656, Italian anatomist and surgeon) who first documented the finding of a human heart being on the right hand side of the body in 1643 [4], and also Matthew Baillie (1761 to 1823, Scottish physician and pathologist) who described the complete mirror image reversal of the thoracic and abdominal organs more than a century later in 1788 [5].

In the following centuries numerous case reports about laterality disorders were published. In a frequently overlooked case study published in 1904, Dr. AK Siewert from Kiev (Ukraine) described a patient ‘who since birth had the unusual combination of symptoms of bronchiectasis and *situs inversus totalis*’ [6]. This was the first description of what has since become known as ‘Kartagener’s syndrome (KS)’ based on the description by Manes Kartagener in 1933 [7], sometimes still referred to as Siewert’s (Zivert’s) syndrome, Siewert(Zivert)-Kartagener syndrome, or ‘immotile cilia syndrome’. It was later renamed ‘primary ciliary dyskinesia (PCD)’ after dysmotile cilia had been observed in individuals diagnosed clinically as having KS [8,9].

Although several decades of research were required to understand the connection behind laterality disorders and the role of cilia, Kartagener was already thinking in the right direction. He noted in one simple sentence that ‘cystic kidneys, which are often compared with congenital bronchiectasis, have been observed in combination with *situs inversus*’ [7,10]. At that time nobody was aware that two types of cilia would be involved in laterality development and that genes causing cystic kidney disease would also play a role [11].

### Structural defects of cilia as the cause for Kartagener’s syndrome

Numerous case reports about laterality defects in humans were published in the following years, but still lacked any direct correlation between laterality disorders and cilia function. During that time, Björn Afzelius had improved sample preparation for electron microscopy, leading to a better preservation and analysis of ciliary ultrastructure [12]. Years later in 1974, Afzelius met Henning Pedersen, who showed Afzelius his unpublished electron micrograph of an immotile human sperm tail lacking dynein arms. Afzelius suggested that Pedersen publish these findings soon. After meeting Pedersen, Afzelius analyzed - together with Rune Eliasson - sperm samples from male patients with stiff, immotile sperm tails. As expected, they also found lack of dynein arms as the cause for immotile human spermatozoa in these patients. Pedersen and Afzelius subsequently published their findings nearly at the same time in 1975 [13-15].

Following these landmark studies, the hypothesis was raised that KS is caused by a genetic lack of dynein arms in cilia [16]. Both Afzelius and Pedersen proved independently that dynein arms were missing in cilia of nasal and bronchial mucosa of affected patients, confirming this hypothesis [17,18]. Afzelius sent a letter to Kartagener to notify him about these findings, but Kartagener’s daughter reported to Afzelius that he had died in August 1975. Evidently this information was never brought to Kartagener’s attention [15,19].

### Special type of cilia at the embryonic organizer

Although the ultrastructural defect causing ciliary immotility and bronchiectasis in patients with KS was identified, the connection between ciliary immotility and body axis development remained elusive. Afzelius early on raised the hypothesis that motile cilia defects can explain a variety of phenotypes/defects observed in patients with KS, but not all. He also discussed the existence of ‘so-called sensory hairs protruding from the cell surface into the extracellular space’ to explain the poor sense of smell and decreased hearing ability in these patients [17]. To explain the connection between cilia and the *situs inversus* observed in patients with KS, Afzelius also mentioned that ‘a further category of cells which carry cilia is the differentiating cells of vertebrate embryos’, referring to a publication analyzing cilia during cardiac development of the chicken [20]. He hypothesized that ‘it is not unreasonable to assume that a malrotation may occur when the ciliary movement causing rotation is lacking’ and that ‘chance alone will determine whether the viscera will take up the normal or the reversed position during embryogenesis, when normal dynein arms are missing’ [17]. In 1976 he noted that the cause of *situs inversus* remains elusive despite many theories, but he also presciently suggested that ‘cilia on the embryonic epithelia have a certain position and fixed beat direction (in normal embryos) and that their beating somehow is instrumental in determining the visceral situs’ [17].

It is unclear whether Afzelius in the early years of his research was aware of experiments performed by numerous embryologists that identified the ‘organizer’ , a group of cells necessary and sufficient to initiate a complex program of spatial organization in competent embryonic tissue (reviewed in [21]). For the groundbreaking research leading to the identification of the organizer performed by Hans Spemann (1869 to 1941) and Hilde Mangold (1898 to 1924), the Nobel Prize in Physiology or Medicine was awarded to Hans Spemann in 1935 [22]. Organizer activity was subsequently also demonstrated in embryos of higher vertebrates such as birds and mammals [23-25] in a structure that Victor Hensen (1835 to 1924) had described in 1876 in an extensive paper and named ‘the node’ , also known as ‘Hensen’s node’ [26].

Nearly 20 years after Afzelius expressed his hypothesis [17], scientists were just starting to put pieces of this puzzle together. It was only in 1994 that Sulik *et al*. published an extensive study on the development of the node and formation of the notochordal plate in embryonic day (E) 7 to 9 mice (Theiler stages 10 to 14). It is of note that Sulik *et al*. still found it important to define several terms such as ‘the rostral end of the murine primitive streak will be referred to as the node (analogous to Hensen’s node in avian species)’ [27] because the structure was not properly assigned in previous studies [28,29]. It is important to note here that the ‘node’ in mouse was renamed several times based on morphological and functional studies. First named ‘archenteron’ by Theiler in 1972 [30] the term ‘node’ was introduced by Beddington in 1991: ‘This (the archenteron) is a misnomer as it is not equivalent to the archenteron in amphibians but, as far as we can tell, corresponds to the dorsal blastopore lip of Xenopus or Hensen’s node of the chick. Therefore, I would suggest that we call it the ‘node” [31]. Later analyses of ciliation and gene expression demonstrated in 2007 that this definition unites two entities, the node and the posterior notochord (PNC), the latter characterized by bi-lateral Nodal expression, motile monocilia and cilia-driven leftward flow and functioning as left-right (LR)-organizer. Using this functional characteristic, the PNC, still commonly named ‘node’ in mouse embryos, corresponds to the gastrocoel roof plate (GRP) and not to the dorsal blastopore lip of amphibians [32]. Nevertheless, in the study of Sulik *et al*. the authors described ‘the presence of groupings of cells in the area of the rostral midline that had small ventral surface areas relative to adjacent cells’ , each with ‘a prominent single, central cilium-like structure on their ventral side’ at the ventral layer of the node [27]. They also performed video microscopy of the node showing that these monocilia cilia were motile, although they failed to find ‘evidence of synchronized activity in time or direction’ [27].

A year later Afzelius, who now interpreted KS as a disease caused by defective or absent cilia, presented five hypotheses to explain the ‘curious’ connection between ciliary defects and loss of laterality control. These hypotheses included combined loss of function of two closely linked genes, one responsible for the asymmetry of viscera and the second responsible for synthesis or assembly of ciliary structures, cytoskeletal defects [33], lack of structural coordination and defective ciliation-or-division switch [34]. As the most likely hypothesis he suggested that ‘there are cilia that have determined positions and a fixed beat direction, much the same as they have on the epidermis of amphibian embryos’ [35] and that ‘ciliary beating in normal embryos is assumed to be instrumental in pushing the heart to the left side, whereas chance alone will determine whether viscera will take up the normal or the reversed position during embryogenesis, when there is no regular ciliary motility’ [17,34]. We can only speculate whether Afzelius had been aware of the study on the development of the node and formation of the notochordal plate presented by Sulik *et al*. [27].

### The nodal signaling cascade and the nodal flow

It still took several years to gain insight into the function of cilia during the process of LR-axis development. Initially, the discovery of molecular networks acting in and around the node during patterning of the body axes constituted the major scientific breakthrough in the analysis of axis development. Although we acknowledge outstanding scientific contributions stemming from research on other model organisms, such as chicken and *Xenopus*, this review will focus on findings obtained in mouse studies unless otherwise stated.

**Figure 1:**
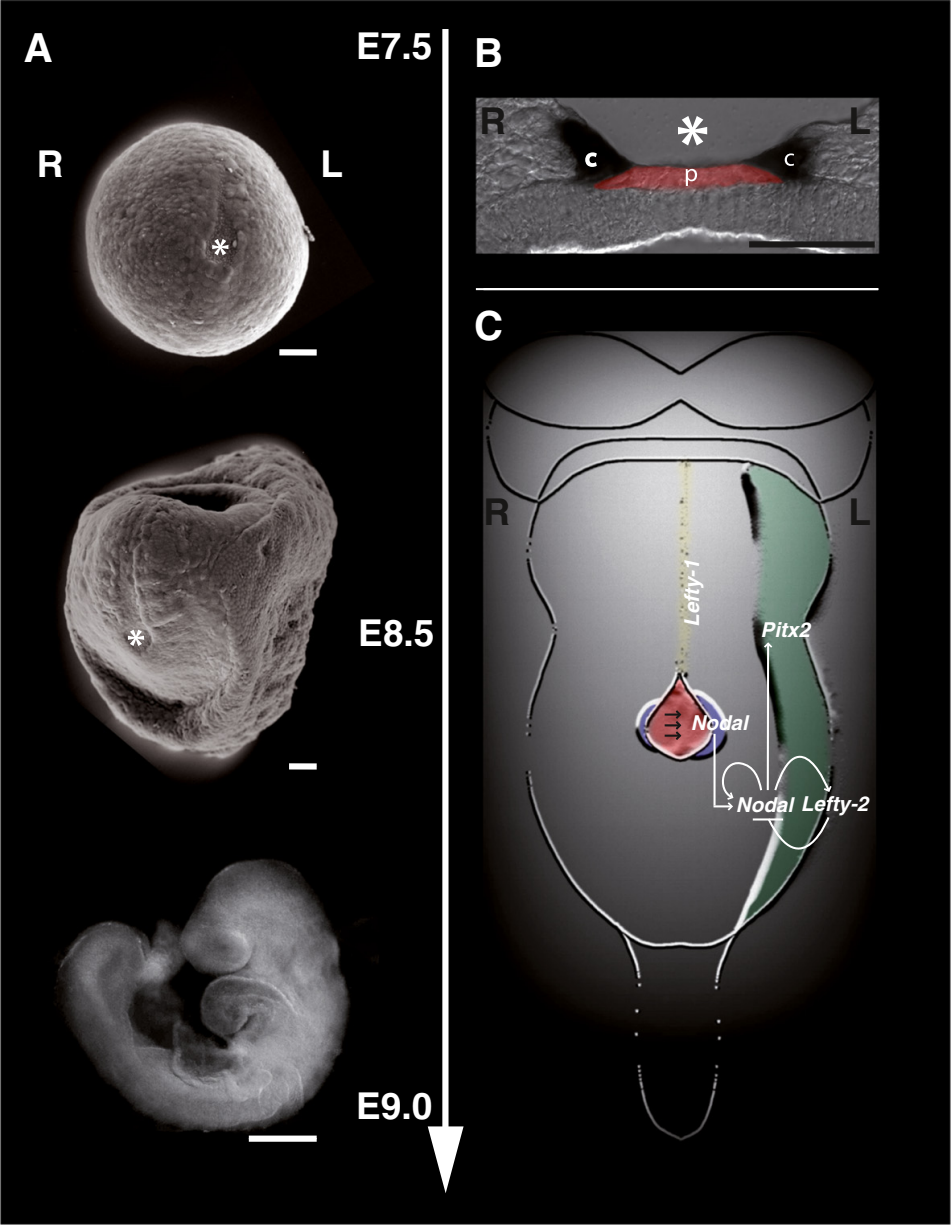
**Timeline for laterality development (A), node structure (B) and genetic cascade of LR patterning (C) in mouse.** LR patterning can be divided into three steps: breakage of symmetry at the node, patterning of the LPM and asymmetric morphogenesis **(A)**. The initial breakage of bilateral symmetry in the mouse occurs in the vicinity of the node (asterisk in **A** and **B**) at the headfold stage corresponding to embryonic day (E)7.5 to E7.8. The node is a transient structure composed of two epithelial layers: the dorsal node and the ventral node [27,123,124]. The ventral node can be separated into the pit region (red in **B** and **C**) and the crown region (marked by *Cerl2* expression (black) in **B)**. Pit cells carry mainly motile monocilia on their apical surface, whereas crown cells mainly carry immotile monocilia [11,118]. Motile monocilia of the node rotate in a clockwise orientation generating a leftward fluid flow over the node cavity (nodal flow; direction is marked by arrows in **C)**. Nodal flow is sensed by cilia of the crown cells and converted into asymmetric signaling involving *Nodal*-*Lefty1*-*Lefty2* regulatory loops in the LPM and induction of Pitx2 expression in the left LPM **(C)**. Key players of this asymmetric signaling cascade are shown with *Nodal* expression in crown cells (purple) and the left LPM (green), *Lefty-2* and *Pitx2* expression in the left LPM (green) and *Lefty-1* expression in the left side of the midline (yellow) [120,123,125-130]. I and II: scanning electron micrographs of wild type mouse embryos (I: 0 somite; II: 5 somites). III: wild type mouse embryo at E9.0 viewed from the left; B: cryo section of a mouse node after in situ hybridization using a *Cerl2* probe photographed using DIC optics. c: crown cells; p: pit cells; R: right; L: left. Scale bars: 50 μm.

In 1993, *Nodal*, one of the key factors of LR-axis development and a member of the transforming growth factor-beta (TGF-beta) superfamily, was identified in mice. *Nodal* expression was detected in a symmetric fashion exclusively at the node or ‘associated with the node’ dependent on the developmental stage analyzed (approximately E7 (primitive streak embryos) to E8.5 (‘coinciding with the disappearance of the node as distinct structure’ [36])) and named *Nodal* due to its localized expression at the node [36]. Surprisingly, three years later, the correlation between asymmetric gene expression and LR-asymmetry in mice became obvious in two independent studies published in *Nature* [37,38]. In these studies, it was demonstrated that expression of *Nodal* was dependent on the developmental stage and in contrast to the previously published study not only in the mouse node [36] but also in the left lateral plate mesoderm (LPM) [37,38]. In the same studies, *Nodal* expression was examined in mouse mutants with perturbed LR-development, especially the *situs inversus viscerum* (*iv*) displaying random LR-asymmetry [39] and the *inversion of embryonic turning* (*inv*) developing *situs inversus* [40]. Both mouse models were extensively used for the analysis of LR-development. It was anticipated that these mutant mice would provide important insights into the understanding of LR-development, although the genetic basis of either mutation was still unknown at that time. The authors demonstrated that *Nodal* expression in these mutants was either normal, reversed or bilateral depending on the morphological LR-asymmetry. This was similar to previous findings in chicken [41] and *Xenopus* [38], thus demonstrating the evolutionary conservation of *Nodal* expression and suggesting that *Nodal* is one of the key regulators of LR-development [37,42]. In the same volume of *Nature* it was shown that another gene named *Lefty* (*left right determination factor*) was transiently and asymmetrically expressed in the left LPM and the left half of the prospective floorplate during LR-axis development. Similar to *Nodal* expression the site of *Lefty* expression correlated with the morphological asymmetry observed in *iv/iv* and *inv/inv* mutants [43]. Extensive studies on *Lefty* function surprisingly demonstrated that the original expression pattern attributed to a single *Lefty* gene in fact derived from two highly-related and chromosomally linked genes, *Lefty-1* and *Lefty-2,* which were both asymmetrically expressed but with distinct expression domains functioning downstream of *iv* and *inv* function [44]. In 1998, the paired-like homeodomain transcription factor 2 (PITX2) was identified in two independent studies as an additional evolutionarily conserved downstream effector of the signaling cascade that establishes asymmetries along the entire LR-axis, the *Nodal* signaling cascade (Figure 1) [45,46].

**Figure 2:**
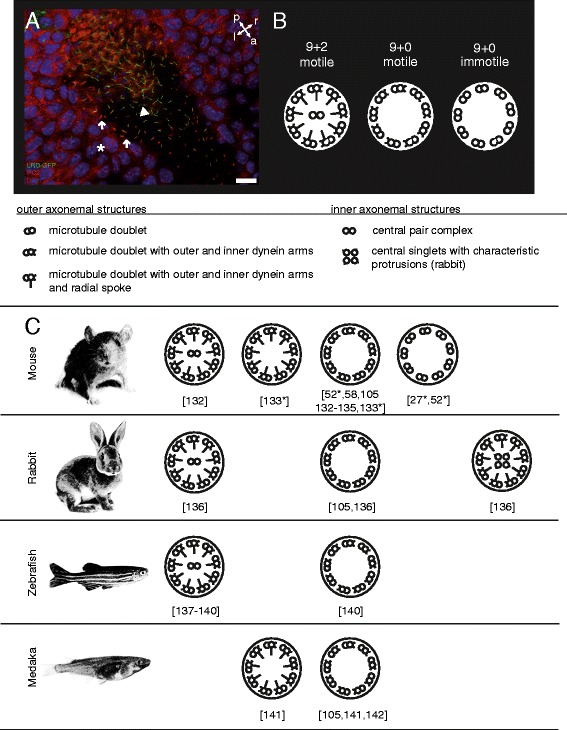
**Types of cilia found at the left-right-organizer of vertebrates.** In mouse, two types of ciliated cells at the node have been demonstrated **(A)**. Pit cells possess motile cilia visualized by a GFP-fusion protein of DNAH11 (LRD-GFP; arrowhead). Most crown cells possess immotile cilia containing polycystin-2 but lacking LRD-GFP (arrow). It is suggested that immotile cilia of crown cells act as flow sensors. Primary cilia containing polycystin-2 are found on endodermal cells adjacent to the node (asterix). [11,101,118]. Classical motile cilia **(B)** possess nine outer microtubule doublets forming a ring around a central pair (CP) of single microtubules, called 9 + 2 axoneme. Outer microtubule doublets possess dynein arms that drive rhythmic movement of the axonemes. Radial spoke complexes project from each outer doublet towards the CP and are thought to be important in regulating motion of the axoneme **(B)**. Motile monocilia **(B)** are found in cells of the LR-organizer in various vertebrates, such as ventral node in mouse, notochordal plate in rabbit or Kupffer’s vesicle in zebrafish and medaka **(C)**. In mouse and medaka, cilia of the LR-organizer usually display the 9 + 0 configuration lacking the CP. In other vertebrates, such as zebrafish, they display 9 + 2 or in rabbit 9 + 0, 9 + 2 and 9 + 4 configuration. Irrespective of the structure, these cilia move in a rotational manner, establishing a leftward-directed fluid flow within the cavity of the LR-organizer. Immotile cilia **(B)** lack motility components such as dynein arms, CP and radial spokes and act as specialized sensors of the cell transducing signals from extracellular stimuli to a cellular response [125,131]. **A**: Immunofluorescent staining detecting polycystin-2 (red) in node monocilia of a 2 somite stage LRD-GFP (green) mouse embryo. **A**: anterior; l: left; p: posterior; r: right. Scale bar: 20 μm [132-142].

At this time it also emerged that cilia function played a role during LR-axis development, and both the *iv* and the *inv* mouse mutants were important in this respect. First described in 1956 [39], *iv* was mapped to mouse chromosome 12 in 1989 [47] and identified by a positional cloning approach in 1997 as an axonemal dynein heavy-chain gene named *left/right-dynein* (*Lrd*, currently known as *dynein, axonemal, heavy chain 11; Dnah11*) [48]. *Lrd* was shown to be expressed in the node of the embryo at E7.5, consistent with having a role in LR-development [48]. Based on the observation that the asymmetric expression patterns of *Nodal* and *Lefty* were randomized in *iv/iv* embryos, it was suggested that *iv* functions early in the genetic hierarchy of LR-specification. Nevertheless, the connection between *Lrd* and cilia at the node was considered unlikely; at that time, it had been supposed that cilia at the node were immotile monocilia lacking dynein arms [49] even though ciliary motility at the node, despite no evidence of synchronized activity in time or direction, had previously been observed [27]. The gene defect underlying the LR-axis defects observed in *inv* mutants was identified a year later in 1998 in two independent studies. These were published five years after the first description of the *inv* phenotype (inversion of embryonic turning and cystic kidneys), and the gene was appropriately named *Inversin* [40,50,51]. Despite these findings, the function of *Inversin* and its connection to axis development remained unknown.

The first tangible evidence in mice that cilia of the node play an important role during LR-development came from the characterization of a mouse mutant lacking the kinesin family member 3b (KIF3B) microtubule-dependent motor protein [52] although it was not the first mouse mutant in which impaired ciliogenesis was reported [53]. Loss of *Kif3b* function resulted in prenatal lethality, neural tube disorganization and randomized LR-asymmetry. *Lefty-2* expression was either bilateral or absent, suggesting - comparable to *iv* and *inv* mutants - that *Kif3b* has a function upstream of the *Nodal* cascade. It was further shown that KIF3B was localized within the axoneme of node monocilia and that *Kif3b* mutant nodes lacked monocilia, suggesting that monocilia of the node play an important role during LR-development. To further elucidate the function of these cilia, video microscopy of the node was performed. In contrast to the then accepted understanding that these cilia lack dynein arms and appear to be immotile [48,49] it was convincingly demonstrated that node monocilia were motile and capable of generating a leftward flow. Based on these data, the authors suggested that motile monocilia at the node generate a directed leftward flow leading to the concentration of a secreted factor to the left side of the node, which then triggers the downstream signaling cascade of left-defining genes, the *Nodal* signaling cascade, still known as the ‘morphogen hypothesis’ [48,52,53].

Aware of the findings of Nonaka *et al.*, Afzelius hypothesized in 1999 that exposure of embryos to highly viscous methyl cellulose during the critical stage would increase the percentage of *situs inversus* [54]. In fact, this hypothesis was confirmed years later using indeed methyl cellulose to influence flow in *Xenopus* and mouse embryos in 2007 and 2012, respectively [55,56].

The finding of nodal flow was a major scientific breakthrough with regard to the connection between cilia function and LR-axis development. Although additional mouse mutants, such as those lacking the KIF3A subunit of kinesin-II, provided supportive evidence for the relevance of cilia at the node during LR-axis development [57,58], it was still necessary to eliminate the last serious doubts.

The first supporting evidence came from an extensive study showing that abnormal nodal flow precedes *situs inversus* in *iv* and *inv* mutant mice, demonstrating immotile cilia in *iv* mutants lacking LRD and a slower net leftward flow in *inv* mutants, due to a more ‘turbulent flow’ despite the fact that cilia lacking Inversin rotate more rapidly [59]. These data suggested that abnormal flow is not the consequence of the abnormal LR-body axis determination but may be its source [59]. The hypothesis that the directed mechanical fluid flow across the node is implicated in the symmetry breaking event was further confirmed by elegant experiments in which wild type embryos and *iv* mutants lacking LRD were cultured under artificial fluid flow conditions. Reversal of laterality was checked by using genetic markers at the LPM, demonstrating randomized or reversed expression previous to organ laterality development, and further confirmed by analysis of organ situs demonstrating reversal of heart looping and embryonic turning [60]. This study convincingly demonstrated that a strong rightward artificial flow could reverse fluid flow at the node and thereby the laterality of wild type embryos, proving that the laterality of treated embryos was successfully controlled by the direction of the artificial flow. Similar experiments were then performed with homozygous *iv/iv* mutant embryos that lack nodal flow due to immotile cilia at the node and exhibit *situs inversus* in half of the mutant embryos. Here, it was demonstrated that *iv/iv* embryos responded even to slow artificial flow, manifesting normal or reversed laterality dependent on the direction of the artificial flow. Although the exact mechanism leading to the initial breakage of symmetry at the node was still unknown, these experiments unequivocally showed that efficient and directed nodal flow generated by motile monocilia at the node was necessary to establish left-right laterality in the embryo (reviewed in [61-63]).

### The laterality-kidney connection

By the year 2000 more than 24 genes involved in axis development had been published, not only by analyses of existing spontaneous mouse mutations but also due to the increasing ability to genetically manipulate the mouse to generate targeted mutations (reviewed in [64]). Nevertheless, knowledge about cilia structure and function at the node was still limited.

Further insight came from a completely unexpected source, namely research performed on autosomal recessive and autosomal dominant polycystic kidney diseases (ARPKD and ADPKD, respectively). Until 1994, several spontaneous mutations in mouse lines had been described resembling ARPKD in humans, namely *cpk* (*congenital polycystic kidneys*) [65] mapped in 1991 to mouse chromosome 12 [66], *bpk* (*BALB/c polycystic kidneys*) [67], *pcy* (*polycystic kidneys*) [68] mapped in 1991 to mouse chromosome 9 [69] and *jck* (*juvenile cystic kidneys*) [70] and others (reviewed in [71]), but none of the disease-causing genes had been identified at that time, making it impossible to study the pathogenesis of PKD in more detail.

Then in 1994, a candidate gene was described to be associated with a mutation causing recessive polycystic kidney disease in mice. This gene was mutated during a large scale mutagenesis program and the line was called TgN(Imorpk)-737Rpw (Imorpk: insertional mutation, Oak Ridge polycystic kidneys), abbreviated *TgN737Rpw* and later on *Oak Ridge Polycystic Kidney* (*orpk*) insertional mutation [72,73]. The targeted allele of this mouse *Tg737* gene, *Tg737*D*2-3*b*Gal*, was published in 2000 [74]. Unlike the original *orpk* allele where all homozygotes survived to birth, embryos homozygous for the *Tg737*D*2-3*b*Gal* mutation arrested in development at mid-gestation and exhibited neural tube defects, enlargement of the pericardial sac and, most notably, LR-asymmetry defects with bilateral expression of *Nodal* and *Lefty-2*. It was shown that nodal monocilia were lacking, similar to the previously described *Kif3b* and *Kif3a* mutants [52,57,58], providing further evidence that motile monocilia at the node are important for proper LR-axis development.

Most interesting with regard to the analysis of both *Tg737* alleles and future analysis of ciliary function during development were the different phenotypes observed in these two *Tg737* alleles: these included cystic kidneys in the hypomorphic *orpk* allele and a more severe phenotype involving motile monocilia at the node in the complete loss-of-function *Tg737*D*2-3*b*Gal* allele. It was suggested that the inability to develop and maintain polarity of renal and node cells as demonstrated by lack of monocilia that were found on virtually every cell of the body [75-77] might be the underlying cause of cyst formation in the kidneys and laterality defects. This observation led to the name ‘Polaris’ for the gene product of *Tg737* [74]. Further analysis demonstrated that Polaris localized just below the apical membrane in the region of the basal bodies and within cilia or flagellar axonemes [78] and that it was important for ciliogenesis of both motile and non-motile primary cilia in different model organisms [79-81], leading to the name ‘*Intraflagellar transport 88*’ (*Ift88*). As a result of these studies, a common key function of cilia in the development of both cystic kidneys and laterality disorders became more apparent.

At that time, based on studies performed in *Caenorhabditis elegans* [82,83], an association between structural and/or functional defects in primary cilia of vertebrate epithelia and another cystic kidney disease caused by mutations in *PKD1* (*ADPKD1*) encoding polycystin-1 [84-87] and *PKD2* (*ADPKD2*) encoding polycystin-2 [88], was suggested.

Indeed, it was possible to show that polycystin-2, next to additional polycystic kidney disease proteins, such as polycystin-1, Polaris and Cystin, localized to primary cilia of the kidney [89,90] supporting this hypothesis. With regard to *Pkd2* mouse mutants, it was previously shown that somatic inactivation of *Pkd2* expectedly resulted in polycystic kidney disease [91] but also prenatal lethality and cardiac defects [92]. Unexpectedly, it was also shown that polycystin-2 was required for LR-axis development and that loss of *Pkd2* function resulted in heterotaxy. Loss of *Nodal*, *Lefty-1* and *Lefty-2* expression and bilateral *Pitx2* expression in *Pkd2* mutants suggested that polycystin-2 is active early during axis development and upstream of the Nodal signaling cascade [93]. The findings of ciliary localization of polycystin-2 in primary cilia of the kidney, LR-axis defects of *Pkd2* mutant mouse embryos and ion channel activity of polycystin-2 [94-96] suggested that polycystin-2 might have a function in cilia of the node, perhaps as an ion channel necessary to sense and translate the leftward flow similar to the proposed function in the kidney.

### The two-cilia model

Just a few months before the involvement of *Pkd2* in laterality development was published, mutations in *DNAH5 (dynein, axonemal, heavy chain 5*) leading to non-functional DNAH5, loss of outer dynein arms and immotile cilia, were published in individuals with PCD and KS [97] and it became increasingly likely that two different types of ciliary defects can cause laterality defects, defects in cilia motility and defects in sensory function.

The next hint came again from the kidney research field showing that polycystin-1 and polycystin-2 mediated mechanosensation in primary cilia of the kidney resulting in calcium influx into the cell [98]. The hypothesis was thus raised that the polycystins might have a similar function at the node.

Only a few months later it was possible to show that two populations of node monocilia initiated LR-asymmetry in the mouse. These populations could be distinguished by LRD, which localized to a motile subset of nodal monocilia, and polycystin-2, which localized to all nodal monocilia although it appeared to be enriched in non-LRD containing cilia (Figure 2) [11]. The same study showed that an asymmetric calcium signaling appeared at the left margin of the node coincident with leftward nodal flow. This signal was absent in both mutant mouse embryos lacking *Lrd* or *Pkd2*. These data suggested that LR-asymmetry is established by an entirely ciliary mechanism consisting of motile, LRD-containing monocilia generating the directed nodal flow and non-motile polycystin-2-containing cilia sensing the flow and initiating an asymmetric calcium signal at the left border of the node, appropriately named the ‘two-cilia model’ [11,99] (and reviewed in [100-103]).

### The polarization of nodal cells

Nevertheless, several questions still remained with regard to ciliary function at the node: 1) how can rotational movement of node monocilia generate the unidirectional flow; 2) how can this unidirectional nodal flow be sensed by the embryo; and 3) what mechanism specifies the differentiation of the two types of cilia at the node?

A theoretical analysis of fluid dynamics at the node proposed a model in which a productive linear flow could result if the rotation axis of the cilia has a posterior tilt [104]. Careful analyses of cilia orientation and cilia movement indeed demonstrated that cilia did not stand perpendicular to the node surface but were tilted posteriorly, confirming the hypothesis postulated by the theoretical analysis [105,106]. That this posterior tilt was necessary to generate a directed flow and that the flow depends on the tilt angle of the cilia was further confirmed using a mechanical model simulating different scenarios [106].

However, the question remained how this posterior tilt of cilia at the node is generated. Establishment of the three body axes, anterior–posterior (AP)-, dorso–ventral (DV)- and LR-axis, is central to the vertebrate body plan. Since the LR-axis is the last axis to be determined during development, LR-polarity must be generated by utilizing the pre-existing positional cues from the AP- and DV-axes.

Previously, it had been shown that complete loss of function of *Biccaudal C* (*BicC*), the gene which is mutated in both the *bpk* mouse model leading to an ARPKD phenotype and the *jcpk* mouse model leading to an ADPKD phenotype [107,108], resulted in LR-axis defects by disrupting the planar alignment of motile cilia required for cilia-driven fluid flow. Furthermore, it had been shown that BICC uncoupled Dishevelled 2 (DVL2) signaling from the canonical Wnt pathway, which has been implicated in antagonizing planar cell polarity (PCP), the orientation of specialized structures within a plane of the epithelial sheet [109]. Thus, it was suggested that establishment of PCP is also involved in the orientation of nodal cilia.

Careful analysis of the basal body orientation demonstrated that the basal bodies of nodal cilia were initially positioned centrally. They then gradually shifted towards the posterior side of the node cells until the majority was located at the posterior side of the ciliated node cells at the two- to three-somite stage, when the velocity of the fluid flow is maximal [110]. Further analysis demonstrated that *Dishevelled* (*Dvl*), a key participant of both canonical and non-canonical Wnt signaling pathways and in mice, represented by three widely expressed and functionally redundant *Dvl* genes (*Dvl1, Dvl2* and *Dvl3*), was relevant for the positioning of basal bodies, confirming this hypothesis [110].

The involvement of the canonical Wnt signaling was further excluded based on analyses performed with embryos deficient in *Wnt3a*, the only ligand that activates the canonical Wnt pathway in the node. These analyses demonstrated that neither the directional flow nor the rotational axis of nodal cilia and position of basal bodies were affected by loss of WNT3A, although *Wnt3a* mutants showed laterality defects [110,111]. On the other hand, blocking RAC1, a small G protein and effector molecule of the non-canonical Wnt pathway, also known as the non-canonical PCP pathway, led to defects in positioning of the basal bodies and vertical nodal flow suggesting that the non-canonical Wnt/PCP pathway is involved [110]. This hypothesis was confirmed by further analyses demonstrating that in the absence of *Vangl1* and *Vangl2*, the two mouse homologues of the *Drosophila* core PCP gene *Van Gogh* (*Vang*) [112] as well as in *Cofilin1;Vangl2* double mutants [113], failure to properly polarize nodal cilia led to randomization of LR-asymmetry (reviewed in [114-116]).

### The sensor of nodal flow

Although mechanistic inside of how leftward nodal flow is generated progressed, exactly how this left side-specific signal gets sensed and transduced remained enigmatic. At that time, only the *Nodal* inhibitor *Cerl2* had been identified to act as a critical target of flow suggesting that symmetry is broken by flow-mediated left-asymmetric release of *Nodal* repression at the midline [117]. Based on the mechanosensory function of polycystin-1 and polycystin-2 in kidney epithelial cells and the elevated left-side specific calcium signal at the node observed in mouse embryos, it was assumed that sensing of this flow occurs through cilia. *Pkd2* was a good candidate since mice lacking polycstin-2 exhibited LR-patterning defects and lost the left-sided expression of *Nodal*, suggesting that *Pkd2* is relevant for cilia function at the node. Interestingly, neither the structure nor the motility of nodal cilia was compromised, suggesting that loss of polycystin-2 in these cilia results in the inability to sense flow [118]. This hypothesis was confirmed by using Ca^2+^ signaling blockers that interfere with polycystin-2 signaling. Rescue experiments demonstrated that although endogenous polycystin-2 localized to cilia of both crown cells and pit cells of the node, the latter located at the central region of the node and mainly possessing motile cilia generating the nodal flow, polycystin-2 was required only in crown cells for the correct establishment of LR-asymmetry. In addition, it was shown that the *Nodal* inhibitor *Cerl2* was not only a critical target of flow but also the major target of *Pkd2* mediated signaling during LR pattern formation. Surprisingly, it was also possible to demonstrate that restoring cilium formation in crown cells of *Kif3a* mutant embryos, which usually completely lack cilia, was also sufficient to induce LR-asymmetry. These data indicated that cilia of the crown cells of the node are the sensors for the leftward fluid generated at the node [118] (and reviewed in [119,120]).

### Motor or sensor?

It was now widely accepted that laterality is initiated at the embryonic LR-organizer, where motile cilia generate leftward flow that is detected by immotile sensory cilia, which then transduce flow into downstream asymmetric signals.

In 2011, *GALNT11* (N-acetylgalactosamine-type O-glycosylation enzyme) was identified as a candidate disease gene in a patient with heterotaxy [121]. Functional analyses performed in *Xenopus tropicalis* demonstrated that galnt11 activated Notch signaling. Live imaging of the cilia of the *Xenopus* organizer was also performed. These analyses demonstrated that either galnt11 or notch1 depletion increased the ratio of motile cilia at the expense of immotile cilia (producing a laterality defect reminiscent of loss of the ciliary sensor polycystin-2) and that *Notch* overexpression decreased this ratio (mimicking the ciliopathy PCD). These data demonstrated that galnt11-mediated notch1 signaling modulates the spatial distribution and ratio of motile and immotile cilia, deciding who is motor and who is sensor at the LR-organizer, a decision which is important for the determination of laterality [122].

## Conclusions

**Figure 3:**
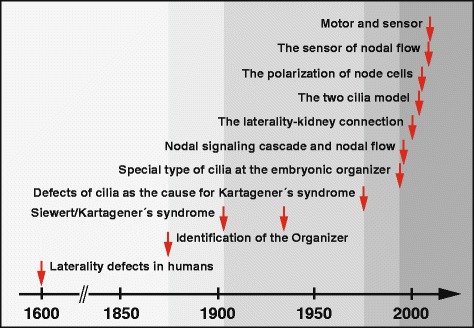
**Milestones of research towards understanding of laterality disorders.** Since the first description of laterality defects around 1600 by Fabrizio several milestones can be identified (marked with red arrows on the time scale) which led to a sharp increase in knowledge with regard to the origin of laterality defects. This included the first description of bronchiectasis in combination with *situs inversus* totalis by Siewert in 1904 [6] and Kartagener in 1933 [7] followed by the demonstration that ciliary defects are the underlying cause of Kartagener’s syndrome in 1976 [17,18]. The demonstration of motile monocilia at the mouse node in 1994 [27] followed by the identification of the first asymmetrically expressed genes in 1996 [37,38], later known as members of the Nodal signaling cascade, opened the wide field of research on the genetic and molecular levels which led to our current knowledge about the connection of *situs inversus* and ciliary abnormalities. It is worth mentioning here that around the same periods, Antony van Leeuwenhoek already in 1675 described a living protozoan ‘provided with diverse incredibly thin feet, or little legs, which were moved very nimbly’ and that the term ‘cilia’ was probably first used by OF Muller in 1786 (reviewed in [143,144]).

This review attempted an historical overview of key publications and experiments that influenced the direction of research and led to our current knowledge connecting the curious link between *situs inversus* and ciliary abnormalities (Figure 3). Of course numerous excellent additional studies exist, which added even more details to this knowledge regarding ciliary structure and function necessary for proper axis development. In addition, a large number of genes influencing node and ciliary structure and function have been identified. Among these, the largest group of genes influences nodal ciliogenesis (ranging from complete absence to short or abnormal cilia) (Additional file 1, Table S1). Another large group of genes influences nodal morphology and shape including orientation of cilia (PCP) necessary to generate the directed leftward flow (Additional file 1, Table S2). A third subset of genes has been shown to cause axonemal defects resulting in dyskinetic cilia and PCD (with or without heterotaxy) (Additional file 1, Table S3). Interestingly, several PCD-causing genes, to the best of our knowledge, do not cause laterality defects in either humans or mouse models but their analyses nonetheless aid to understand the structure of nodal cilia (Additional file 1, Table S4).

We can only speculate how Bjorn Afzelius, who died April 27 2008, would view the tremendous advances in molecular and genetic research that have convincingly linked ciliary function at the node to LR-axis development and, amazingly, confirmed his hypothesis from twenty years before.

We can anticipate that combined efforts by clinicians and basic researchers as well as the brisk pace of advancements in genetic analyses and modification of animal models will bring even greater understanding of how ciliary function influences LR-axis development and we are looking forward to the advancements in this field.

## Additional file

Additional file 1: Table S1–S4. dummyonly

## Abbreviations

ADPKD: autosomal dominant polycystic kidney disease
AP: anterior-posterior
ARPKD: autosomal recessive polycystic kidney disease
*BicC*: Biccaudal C
*Bpk*: BALB/c polycystic kidneys
*Cpk*: Congenital polycystic kidneys
*Dnah11*: Dynein, axonemal, heavy chain 11
*DNAH5*: Dynein, axonemal, heavy chain 5
DV: dorso-ventral
*Dvl*: Disheveled
E: embryonic day
GALNT11: N-acetylgalactosamine-type O-glycosylation enzyme
GFP: green fluorescent protein
GRP: gastrocoel roof plate
*Ift88*: Intraflagellar transport 88
*Inv*: Inversion of embryonic turning
*Iv*: Situs inversus viscerum
*Jck*: Juvenile cystic kidneys
KIF3A: Kinesin family member 3A
KIF3B: Kinesin family member 3B
KS: Kartagener’s syndrome
LPM: lateral plate mesoderm
LR: left-right
*Lrd*: Left/right-dynein
*Orpk*: Oak Ridge Polycystic Kidney
PCD: primary ciliary dyskinesia
PCP: planar cell polarity
*pcy*: Polycystic kidneys
*Pitx2*: Paired-like homeodomain transcription factor 2
PKD: polycystic kidney disease
*PKD1*: Polycystic kidney disease gene 1
*PKD2*: Polycystic kidney disease gene 2
PNC: posterior notched
RAC1: RAS-related C3 botulinum substrate 1
TGF: transforming growth factor
*Vangl*: Van Gogh like
Wnt: Wingless-type MMTV integration site family
