# Supplementary material for: Situs inversus and ciliary abnormalities: 20 years later, what is the connection?
Source: Cilia. 2015 Jan 14;4:1. doi: 10.1186/s13630-014-0010-9 (PMC4292827; doi:10.1186/s13630-014-0010-9)
Supplement: Additional file 1: Table S1–S4. — dummyonly [file 13630_2014_10_MOESM1_ESM.docx]

**Situs inversus and ciliary abnormalities: 20 years later, what is the connection?**

Petra Pennekamp^1#^, Tabea Menchen^1^, Bernd Dworniczak^2^, Hiroshi Hamada^3^

**Supplementary Tables**

**Supplementary table 1**: Genes that have been shown to influence ciliogenesis at the node in mice, ranging from complete absence to short or abnormal cilia.

| ***Gene*** | **full name** | **References** |
| --- | --- | --- |
| *Acvr1* | activin A receptor, type 1 | [1,2] |
| *Arl13b* | ADP-ribosylation factor-like 13B | [3,4] |
| *Atmin* | ATM interactor | [5] |
| *B9d1* | B9 protein domain 1 | [6] |
| *B9d2* | B9 protein domain 2 | [6,7] |
| *Cluap1* | clusterin associated protein 1 | [8] |
| *Foxj1* | forkhead box J1 | [9,10] |
| *Ift57* | intraflagellar transport 57 | [11] |
| *Ift88* | intraflagellar transport 88 | [12,13] |
| *Ift122* | intraflagellar transport 122 | [14] |
| *Ift172* | intraflagellar transport 172 | [15,16] |
| *Intu* | inturned planar cell polarity effector homolog (Drosophila) | [17] |
| *Kif3a* | kinesin family member 3A | [18,19] |
| *Kif3b* | kinesin family member 3B | [20] |
| *Mks1* | Meckel syndrome, type 1 | [21] |
| *Noto* | notochord homolog (Xenopus laevis) | [22] |
| *Ofd1* | oral-facial-digital syndrome 1 gene homolog (human) | [23] |
| *Pifo* | primary cilia formation | [24] |
| *Rfx3* | regulatory factor X, 3 (influences HLA class II expression) | [25] |
| *Rpgrip1* | Rpgrip1-like | [26] |
| *T* | brachyury | [27] |
| *Talpid3* | RIKEN cDNA 2700049A03 gene | [28-30] |
| *Tbx6* | T-box 6 | [31] |
| *Zic2* | zinc finger protein of the cerebellum 2 | [32] |

**Supplementary table 2**: Genes that have been shown to influence node morphology and shape including orientation of cilia (PCP) which is necessary to generate directed leftward flow in mice.

| ***Gene*** | **full name** | **References** |
| --- | --- | --- |
| *Bicc1* | bicaudal C homolog 1 (Drosophila) | [33,34] |
| *Dll1* | delta-like 1 (Drosophila) | [35,36] |
| *Dvl1 Dvl2 Dvl3* | dishevelled, dsh homolog 1 (Drosophila) dishevelled 2, dsh homolog (Drosophila) dishevelled 3, dsh homolog (Drosophila) | [37] |
| *Cfl1 + Vangl 2* | cofilin 1, non-muscle + vang-like 2 (van gogh, Drosophila) | [38] |
| *Ednrb* | endothelin receptor type B | [39] |
| *Epb4.1l5* | erythrocyte protein band 4.1-like 5 | [40] |
| *Fn1* | fibronectin 1 | [41] |
| *Foxa2* | forkhead box A2 | [42,43] |
| *Invs* | inversin | [44,45] |
| *Notch1 + Notch2* | notch 1 + notch2 | [35] |
| *Smarcd3* | SWI/SNF related, matrix associated, actin dependent regulator of chromatin, subfamily d, member 3 | [46] |
| *Sufu* | suppressor of fused homolog (Drosophila) | [47] |
| *Vangl1+Vangl2* | vang-like 1 (van gogh, Drosophila) +  vang-like 2 (van gogh, Drosophila) | [38] |

**Supplementary table 3**: Genes that have been shown to cause axonemal defects resulting in dyskinetic cilia and PCD in with or without heterotaxy in humans (and mice).

| ***Genes encoding subunits of axonemal outer dynein arm components*** | | |
| --- | --- | --- |
| ***Gene*** | **full name** | **References** |
| *DNAH5/Dnah5* | dynein, axonemal, heavy chain 5 | [48-51] |
| *DNAH11/Dnah11* | dynein, axonemal, heavy chain 11 | [51-53] |
| *DANI1/Dnaic1* | dynein, axonemal, intermediate chain 1 | [51,54-56] |
| *DNAI2* | dynein, axonemal, intermediate chain 2 | [57] |
| *DNAL1* | dynein, axonemal, light chain 1 | [58] |
| *NME8* | NME/NM23 family member 8 | [59] |
|  | | |
| ***Genes encoding for proteins required for the docking of ODA complexes*** | | |
| ***Gene*** | **full name** | **References** |
| *ARMC4/Armc4* | armadillo repeat containing 4 | [51,60] |
| *CCDC114* | coiled-coil domain containing 114 | [61] |
| *CCDC151/Ccdc151* | coiled-coil domain containing 151 | [62] |
|  | | |
| ***Genes encoding for proteins required for pre-assembly of dynein arm components*** | | |
| ***Gene*** | **full name** | **References** |
| *C21ORF59* | RIKEN cDNA 1110004E09 gene (human homolog: Chomosome 21 open reading frame 59) | [63] |
| *CCDC103* | coiled-coil domain containing 103 | [64] |
| *DNAAF1* | dynein, axonemal assembly factor 1 | [65] |
| *DNAAF2/Dnaaf2* | dynein, axonemal assembly factor 2 | [66,67] |
| *DNAAF3/Dnaaf3* | dynein, axonemal assembly factor 3 | [51,68] |
| *DYX1C1/Dyx1c1* | dyslexia susceptibility 1 candidate 1 | [51,69] |
| *HEATR2* | HEAT repeat containing 2 | [70] |
| *LLRC6* | leucine rich repeat containing 6 | [71] |
| *SPAG1* | sperm associated antigen 1 | [72] |
| *ZMYND10* | zinc finger, MYND-type containing 10 | [73] |
|  | | |
| ***Genes influencing IDAs and nexin-dynein regulatory complexes (N-DRCs) and microtubular organization*** | | |
| ***Gene*** | **full name** | **References** |
| *CCDC39* | coiled-coil domain containing 39 | [51,74] |
| *CCDC40* | coiled-coil domain containing 40 | [75] |
|  | | |
| ***Genes encoding for N-DRC components **** | | |
| ***Gene*** | **full name** | **References** |
| *CCDC65* | coiled-coil domain containing 65 | [63] |
| *DRC1/Drc1* | dynein regulatory complex subunit 1 | [51,76] |

### *For *CCDC65* and *DRC1/Drc1* so far no human laterality defects have been reported. But, for *Drc1* a mouse mutant was identified by the Bench to Bassinet Program (B2B/CVDC) showing situs inversus totalis (*Drc1b2b2237Clo* [51]) suggesting that these genes are also important for cilia motility at the node.

**Supplementary table 4**: Genes that have been shown to cause PCD or RGMC. Laterality defects have not been observed so far in humans and mouse mutants.

| ***Genes encoding for radial spoke proteins*** | | |
| --- | --- | --- |
| ***Gene*** | **full name** | **References** |
| *RSPH1/Rsph1* | radial spoke head 1 homolog (Chlamydomonas) | [77-79] |
| *RSPH4* | radial spoke head 4 homolog (Chlamydomonas) | [78,80] |
| *RSPH9* | radial spoke head 9 homolog (Chlamydomonas) | [78-80] |
|  | | |
| ***Gene encoding for central pair complex components*** | | |
| ***Gene*** | **full name** | **References** |
| *HYDIN/Hydin** | HYDIN, axonemal central pair apparatus protein | [81-83] |
|  | | |
| ***Genes causing reduced generation of multiple motile cilia (RGMC)*** | | |
| ***Gene*** | **full name** | **References** |
| *CCNO* | cyclin O | [84] |
| *MCIDAS* | multiciliate differentiation and DNA synthesis associated cell cycle protein | [85] |

**HYDIN/Hydin* encodes for a subunit of the central pair complex [82,83]. Lack of expression at the node and lack of laterality defects in these mutants provide high evidence that central pairs are not relevant for ciliary function during LR-development despite that fact that central pairs can be observed in nodal monocilia [86] (Figure 2).

### Supplementary References

[1] Kishigami S, Yoshikawa S, Castranio T, Okazaki K, Furuta Y, Mishina Y: **BMP signaling through ACVRI is required for left-right patterning in the early mouse embryo.** *Dev Biol* 2004**, 276**:185-193.

[2] Komatsu Y, Kaartinen V, Mishina Y: **Cell cycle arrest in node cells governs ciliogenesis at the node to break left-right symmetry.** *Development* 2011**, 138**:3915-3920.

[3] Caspary T, Larkins CE, Anderson KV: **The graded response to Sonic Hedgehog depends on cilia architecture.** *Dev Cell* 2007**, 12**:767-778.

[4] Larkins CE, Long AB, Caspary T: **Defective Nodal and Cerl2 expression in the Arl13b(hnn) mutant node underlie its heterotaxia.** *Dev Biol* 2012**, 367**:15-24.

[5] Ermakov A, Stevens JL, Whitehill E, Robson JE, Pieles G, Brooker D, Goggolidou P, Powles-Glover N, Hacker T, Young SR, Dear N, Hirst E, Tymowska-Lalanne Z, Briscoe J, Bhattacharya S, Norris DP: **Mouse mutagenesis identifies novel roles for left-right patterning genes in pulmonary, craniofacial, ocular, and limb development.** *Dev Dyn* 2009**, 238**:581-594.

[6] Dowdle WE, Robinson JF, Kneist A, Sirerol-Piquer MS, Frints SG, Corbit KC, Zaghloul NA, van Lijnschoten G, Mulders L, Verver DE, Zerres K, Reed RR, Attie-Bitach T, Johnson CA, Garcia-Verdugo JM, Katsanis N, Bergmann C, Reiter JF: **Disruption of a ciliary B9 protein complex causes Meckel syndrome.** *Am J Hum Genet* 2011**, 89**:94-110.

[7] Town T, Breunig JJ, Sarkisian MR, Spilianakis C, Ayoub AE, Liu X, Ferrandino AF, Gallagher AR, Li MO, Rakic P, Flavell RA: **The stumpy gene is required for mammalian ciliogenesis.** *Proc Natl Acad Sci U S A* 2008**, 105**:2853-2858.

[8] Botilde Y, Yoshiba S, Shinohara K, Hasegawa T, Nishimura H, Shiratori H, Hamada H: **Cluap1 localizes preferentially to the base and tip of cilia and is required for ciliogenesis in the mouse embryo.** *Dev Biol* 2013**, 381**:203-212.

[9] Brody SL, Yan XH, Wuerffel MK, Song SK, Shapiro SD: **Ciliogenesis and left-right axis defects in forkhead factor HFH-4-null mice.** *Am J Respir Cell Mol Biol* 2000**, 23**:45-51.

[10] Zhang M, Bolfing MF, Knowles HJ, Karnes H, Hackett BP: **Foxj1 regulates asymmetric gene expression during left-right axis patterning in mice.** *Biochem Biophys Res Commun* 2004**, 324**:1413-1420.

[11] Houde C, Dickinson RJ, Houtzager VM, Cullum R, Montpetit R, Metzler M, Simpson EM, Roy S, Hayden MR, Hoodless PA, Nicholson DW: **Hippi is essential for node cilia assembly and Sonic hedgehog signaling.** *Dev Biol* 2006**, 300**:523-533.

[12] Murcia NS, Richards WG, Yoder BK, Mucenski ML, Dunlap JR, Woychik RP: **The Oak Ridge Polycystic Kidney (orpk) disease gene is required for left-right axis determination.** *Development* 2000**, 127**:2347-2355.

[13] Wheatley D: **Primary cilia in normal and pathological tissues.** *Pathobiology* 1995**, 63**:222-238.

[14] Cortellino S, Wang C, Wang B, Bassi MR, Caretti E, Champeval D, Calmont A, Jarnik M, Burch J, Zaret KS, Larue L, Bellacosa A: **Defective ciliogenesis, embryonic lethality and severe impairment of the Sonic Hedgehog pathway caused by inactivation of the mouse complex A intraflagellar transport gene Ift122/Wdr10, partially overlapping with the DNA repair gene Med1/Mbd4.** *Dev Biol* 2009**, 325**:225-237.

[15] Huangfu D, Liu A, Rakeman AS, Murcia NS, Niswander L, Anderson KV: **Hedgehog signalling in the mouse requires intraflagellar transport proteins.** *Nature* 2003**, 426**:83-87.

[16] Friedland-Little JM, Hoffmann AD, Ocbina PJ, Peterson MA, Bosman JD, Chen Y, Cheng SY, Anderson KV, Moskowitz IP: **A novel murine allele of Intraflagellar Transport Protein 172 causes a syndrome including VACTERL-like features with hydrocephalus.** *Hum Mol Genet* 2011**, 20**:3725-3737.

[17] Zeng H, Hoover AN, Liu A: **PCP effector gene Inturned is an important regulator of cilia formation and embryonic development in mammals.** *Dev Biol* 2010**, 339**:418-428.

[18] Marszalek JR, Ruiz-Lozano P, Roberts E, Chien KR, Goldstein LS: **Situs inversus and embryonic ciliary morphogenesis defects in mouse mutants lacking the KIF3A subunit of kinesin-II.** *Proc Natl Acad Sci U S A* 1999**, 96**:5043-5048.

[19] Takeda S, Yonekawa Y, Tanaka Y, Okada Y, Nonaka S, Hirokawa N: **Left-right asymmetry and kinesin superfamily protein KIF3A: new insights in determination of laterality and mesoderm induction by kif3A-/- mice analysis.** *J Cell Biol* 1999**, 145**:825-836.

[20] Nonaka S, Tanaka Y, Okada Y, Takeda S, Harada A, Kanai Y, Kido M, Hirokawa N: **Randomization of left-right asymmetry due to loss of nodal cilia generating leftward flow of extraembryonic fluid in mice lacking KIF3B motor protein.** *Cell* 1998**, 95**:829-837.

[21] Cui C, Chatterjee B, Francis D, Yu Q, SanAgustin JT, Francis R, Tansey T, Henry C, Wang B, Lemley B, Pazour GJ, Lo CW: **Disruption of Mks1 localization to the mother centriole causes cilia defects and developmental malformations in Meckel-Gruber syndrome.** *Dis Model Mech* 2011**, 4**:43-56.

[22] Beckers A, Alten L, Viebahn C, Andre P, Gossler A: **The mouse homeobox gene Noto regulates node morphogenesis, notochordal ciliogenesis, and left right patterning.** *Proc Natl Acad Sci U S A* 2007**, 104**:15765-15770.

[23] Ferrante MI, Zullo A, Barra A, Bimonte S, Messaddeq N, Studer M, Dolle P, Franco B: **Oral-facial-digital type I protein is required for primary cilia formation and left-right axis specification.** *Nat Genet* 2006**, 38**:112-117.

[24] Kinzel D, Boldt K, Davis EE, Burtscher I, Trumbach D, Diplas B, Attie-Bitach T, Wurst W, Katsanis N, Ueffing M, Lickert H: **Pitchfork regulates primary cilia disassembly and left-right asymmetry.** *Dev Cell* 2010**, 19**:66-77.

[25] Bonnafe E, Touka M, AitLounis A, Baas D, Barras E, Ucla C, Moreau A, Flamant F, Dubruille R, Couble P, Collignon J, Durand B, Reith W: **The transcription factor RFX3 directs nodal cilium development and left-right asymmetry specification.** *Mol Cell Biol* 2004**, 24**:4417-4427.

[26] Vierkotten J, Dildrop R, Peters T, Wang B, Ruther U: **Ftm is a novel basal body protein of cilia involved in Shh signalling.** *Development* 2007**, 134**:2569-2577.

[27] Concepcion D, Papaioannou VE: **Nature and extent of left/right axis defects in T(Wis) /T(Wis) mutant mouse embryos.** *Dev Dyn* 2014**, 243**:1046-1053.

[28] Yin Y, Bangs F, Paton IR, Prescott A, James J, Davey MG, Whitley P, Genikhovich G, Technau U, Burt DW, Tickle C: **The Talpid3 gene (KIAA0586) encodes a centrosomal protein that is essential for primary cilia formation.** *Development* 2009**, 136**:655-664.

[29] Bangs F, Antonio N, Thongnuek P, Welten M, Davey MG, Briscoe J, Tickle C: **Generation of mice with functional inactivation of talpid3, a gene first identified in chicken.** *Development* 2011**, 138**:3261-3272.

[30] Kobayashi T, Kim S, Lin YC, Inoue T, Dynlacht BD: **The CP110-interacting proteins Talpid3 and Cep290 play overlapping and distinct roles in cilia assembly.** *J Cell Biol* 2014**, 204**:215-229.

[31] Hadjantonakis AK, Pisano E, Papaioannou VE: **Tbx6 regulates left/right patterning in mouse embryos through effects on nodal cilia and perinodal signaling.** *PLoS One* 2008**, 3**:e2511.

[32] Barratt KS, Glanville-Jones HC, Arkell RM: **The Zic2 gene directs the formation and function of node cilia to control cardiac situs.** *Genesis* 2014**, 52**:626-635.

[33] Maisonneuve C, Guilleret I, Vick P, Weber T, Andre P, Beyer T, Blum M, Constam DB: **Bicaudal C, a novel regulator of Dvl signaling abutting RNA-processing bodies, controls cilia orientation and leftward flow.** *Development* 2009**, 136**:3019-3030.

[34] Wessely O, Tran U, Zakin L, De Robertis EM: **Identification and expression of the mammalian homologue of Bicaudal-C.** *Mech Dev* 2001**, 101**:267-270.

[35] Krebs LT, Iwai N, Nonaka S, Welsh IC, Lan Y, Jiang R, Saijoh Y, O'Brien TP, Hamada H, Gridley T: **Notch signaling regulates left-right asymmetry determination by inducing Nodal expression.** *Genes Dev* 2003**, 17**:1207-1212.

[36] Przemeck GK, Heinzmann U, Beckers J, Hrabe de Angelis M: **Node and midline defects are associated with left-right development in Delta1 mutant embryos.** *Development* 2003**, 130**:3-13.

[37] Hashimoto M, Shinohara K, Wang J, Ikeuchi S, Yoshiba S, Meno C, Nonaka S, Takada S, Hatta K, Wynshaw-Boris A, Hamada H: **Planar polarization of node cells determines the rotational axis of node cilia.** *Nat Cell Biol* 2010**, 12**:170-176.

[38] Mahaffey JP, Grego-Bessa J, Liem KF,Jr, Anderson KV: **Cofilin and Vangl2 cooperate in the initiation of planar cell polarity in the mouse embryo.** *Development* 2013**, 140**:1262-1271.

[39] Welsh IC, O'Brien TP: **Loss of late primitive streak mesoderm and interruption of left-right morphogenesis in the Ednrb(s-1Acrg) mutant mouse.** *Dev Biol* 2000**, 225**:151-168.

[40] Lee JD, Migeotte I, Anderson KV: **Left-right patterning in the mouse requires Epb4.1l5-dependent morphogenesis of the node and midline.** *Dev Biol* 2010**, 346**:237-246.

[41] Pulina MV, Hou SY, Mittal A, Julich D, Whittaker CA, Holley SA, Hynes RO, Astrof S: **Essential roles of fibronectin in the development of the left-right embryonic body plan.** *Dev Biol* 2011**, 354**:208-220.

[42] Ang SL, Rossant J: **HNF-3 beta is essential for node and notochord formation in mouse development.** *Cell* 1994**, 78**:561-574.

[43] Weinstein DC, Ruiz i Altaba A, Chen WS, Hoodless P, Prezioso VR, Jessell TM, Darnell JE,Jr: **The winged-helix transcription factor HNF-3 beta is required for notochord development in the mouse embryo.** *Cell* 1994**, 78**:575-588.

[44] Okada Y, Nonaka S, Tanaka Y, Saijoh Y, Hamada H, Hirokawa N: **Abnormal nodal flow precedes situs inversus in iv and inv mice.** *Mol Cell* 1999**, 4**:459-468.

[45] Watanabe D, Saijoh Y, Nonaka S, Sasaki G, Ikawa Y, Yokoyama T, Hamada H: **The left-right determinant Inversin is a component of node monocilia and other 9+0 cilia.** *Development* 2003**, 130**:1725-1734.

[46] Takeuchi JK, Lickert H, Bisgrove BW, Sun X, Yamamoto M, Chawengsaksophak K, Hamada H, Yost HJ, Rossant J, Bruneau BG: **Baf60c is a nuclear Notch signaling component required for the establishment of left-right asymmetry.** *Proc Natl Acad Sci U S A* 2007**, 104**:846-851.

[47] Cooper AF, Yu KP, Brueckner M, Brailey LL, Johnson L, McGrath JM, Bale AE: **Cardiac and CNS defects in a mouse with targeted disruption of suppressor of fused.** *Development* 2005**, 132**:4407-4417.

[48] Olbrich H, Haffner K, Kispert A, Volkel A, Volz A, Sasmaz G, Reinhardt R, Hennig S, Lehrach H, Konietzko N, Zariwala M, Noone PG, Knowles M, Mitchison HM, Meeks M, Chung EM, Hildebrandt F, Sudbrak R, Omran H: **Mutations in DNAH5 cause primary ciliary dyskinesia and randomization of left-right asymmetry.** *Nat Genet* 2002**, 30**:143-144.

[49] Ibanez-Tallon I, Gorokhova S, Heintz N: **Loss of function of axonemal dynein Mdnah5 causes primary ciliary dyskinesia and hydrocephalus.** *Hum Mol Genet* 2002**, 11**:715-721.

[50] Ibanez-Tallon I, Pagenstecher A, Fliegauf M, Olbrich H, Kispert A, Ketelsen UP, North A, Heintz N, Omran H: **Dysfunction of axonemal dynein heavy chain Mdnah5 inhibits ependymal flow and reveals a novel mechanism for hydrocephalus formation.** *Hum Mol Genet* 2004**, 13**:2133-2141.

[51] Lo C: **Information submitted by the NHLBI Cardiovascular Development Consortium (CvDC), Bench to Bassinet Program.** *MGI Direct Data Submission (B2B/CvDC)*.

[52] Schwabe GC, Hoffmann K, Loges NT, Birker D, Rossier C, de Santi MM, Olbrich H, Fliegauf M, Failly M, Liebers U, Collura M, Gaedicke G, Mundlos S, Wahn U, Blouin JL, Niggemann B, Omran H, Antonarakis SE, Bartoloni L: **Primary ciliary dyskinesia associated with normal axoneme ultrastructure is caused by DNAH11 mutations.** *Hum Mutat* 2008**, 29**:289-298.

[53] Knowles MR, Leigh MW, Carson JL, Davis SD, Dell SD, Ferkol TW, Olivier KN, Sagel SD, Rosenfeld M, Burns KA, Minnix SL, Armstrong MC, Lori A, Hazucha MJ, Loges NT, Olbrich H, Becker-Heck A, Schmidts M, Werner C, Omran H, Zariwala MA, for the Genetic Disorders of Mucociliary Clearance Consortium: **Mutations of DNAH11 in patients with primary ciliary dyskinesia with normal ciliary ultrastructure.** *Thorax* 2011.

[54] Pennarun G, Escudier E, Chapelin C, Bridoux AM, Cacheux V, Roger G, Clement A, Goossens M, Amselem S, Duriez B: **Loss-of-function mutations in a human gene related to Chlamydomonas reinhardtii dynein IC78 result in primary ciliary dyskinesia.** *Am J Hum Genet* 1999**, 65**:1508-1519

[55] ] Guichard C, Harricane MC, Lafitte JJ, Godard P, Zaegel M, Tack V, Lalau G, Bouvagnet P: **Axonemal dynein intermediate-chain gene (DNAI1) mutations result in situs inversus and primary ciliary dyskinesia (Kartagener syndrome).** *Am J Hum Genet* 2001**, 68**:1030-1035.

[56] Ostrowski LE, Yin W, Rogers TD, Busalacchi KB, Chua M, O'Neal WK, Grubb BR: **Conditional deletion of dnaic1 in a murine model of primary ciliary dyskinesia causes chronic rhinosinusitis.** *Am J Respir Cell Mol Biol* 2010**, 43**:55-63.

[57] Loges NT, Olbrich H, Fenske L, Mussaffi H, Horvath J, Fliegauf M, Kuhl H, Baktai G, Peterffy E, Chodhari R, Chung EM, Rutman A, O'Callaghan C, Blau H, Tiszlavicz L, Voelkel K, Witt M, Zietkiewicz E, Neesen J, Reinhardt R, Mitchison HM, Omran H: **DNAI2 mutations cause primary ciliary dyskinesia with defects in the outer dynein arm.** *Am J Hum Genet* 2008**, 83**:547-558.

[58] Mazor M, Alkrinawi S, Chalifa-Caspi V, Manor E, Sheffield VC, Aviram M, Parvari R: **Primary ciliary dyskinesia caused by homozygous mutation in DNAL1, encoding dynein light chain 1.** *Am J Hum Genet* 2011**, 88**:599-607.

[59] Duriez B, Duquesnoy P, Escudier E, Bridoux AM, Escalier D, Rayet I, Marcos E, Vojtek AM, Bercher JF, Amselem S: **A common variant in combination with a nonsense mutation in a member of the thioredoxin family causes primary ciliary dyskinesia.** *Proc Natl Acad Sci U S A* 2007**, 104**:3336-3341.

[60] Hjeij R, Lindstrand A, Francis R, Zariwala MA, Liu X, Li Y, Damerla R, Dougherty GW, Abouhamed M, Olbrich H, Loges NT, Pennekamp P, Davis EE, Carvalho CM, Pehlivan D, Werner C, Raidt J, Kohler G, Haffner K, Reyes-Mugica M, Lupski JR, Leigh MW, Rosenfeld M, Morgan LC, Knowles MR, Lo CW, Katsanis N, Omran H: **ARMC4 mutations cause primary ciliary dyskinesia with randomization of left/right body asymmetry.** *Am J Hum Genet* 2013**, 93**:357-367.

[61] Knowles MR, Leigh MW, Ostrowski LE, Huang L, Carson JL, Hazucha MJ, Yin W, Berg JS, Davis SD, Dell SD, Ferkol TW, Rosenfeld M, Sagel SD, Milla CE, Olivier KN, Turner EH, Lewis AP, Bamshad MJ, Nickerson DA, Shendure J, Zariwala MA, Genetic Disorders of Mucociliary Clearance Consortium: **Exome sequencing identifies mutations in CCDC114 as a cause of primary ciliary dyskinesia.** *Am J Hum Genet* 2013**, 92**:99-106.

[62] Hjeij R, Onoufriadis A, Watson CM, Slagle CE, Klena NT, Dougherty GW, Kurkowiak M, Loges NT, Diggle CP, Morante NF, Gabriel GC, Lemke KL, Li Y, Pennekamp P, Menchen T, Konert F, Marthin JK, Mans DA, Letteboer SJ, Werner C, Burgoyne T, Westermann C, Rutman A, Carr IM, O'Callaghan C, Moya E, Chung EM; UK10K Consortium, Sheridan E, Nielsen KG, Roepman R, Bartscherer K, Burdine RD, Lo CW, Omran H, Mitchison HM: **CCDC151 mutations cause primary ciliary dyskinesia by disruption of the outer dynein arm docking complex formation**. Am J Hum Genet 2014, **95**:257-274.[See comment in PubMed Commons below](http://www.ncbi.nlm.nih.gov/pubmed/25192045#comments)

[63] Austin-Tse C, Halbritter J, Zariwala MA, Gilberti RM, Gee HY, Hellman N, Pathak N, Liu Y, Panizzi JR, Patel-King RS, Tritschler D, Bower R, O'Toole E, Porath JD, Hurd TW, Chaki M, Diaz KA, Kohl S, Lovric S, Hwang DY, Braun DA, Schueler M, Airik R, Otto EA, Leigh MW, Noone PG, Carson JL, Davis SD, Pittman JE, Ferkol TW, Atkinson JJ, Olivier KN, Sagel SD, Dell SD, Rosenfeld M, Milla CE, Loges NT, Omran H, Porter ME, King SM, Knowles MR, Drummond IA, Hildebrandt F: **Zebrafish Ciliopathy Screen Plus Human Mutational Analysis Identifies C21orf59 and CCDC65 Defects as Causing Primary Ciliary Dyskinesia.** *Am J Hum Genet* 2013**, 93**:672-686.

[64] Panizzi JR, Becker-Heck A, Castleman VH, Al-Mutairi DA, Liu Y, Loges NT, Pathak N, Austin-Tse C, Sheridan E, Schmidts M, Olbrich H, Werner C, Haffner K, Hellman N, Chodhari R, Gupta A, Kramer-Zucker A, Olale F, Burdine RD, Schier AF, O'Callaghan C, Chung EM, Reinhardt R, Mitchison HM, King SM, Omran H, Drummond IA: **CCDC103 mutations cause primary ciliary dyskinesia by disrupting assembly of ciliary dynein arms.** *Nat Genet* 2012.

[65] Loges NT, Olbrich H, Becker-Heck A, Haffner K, Heer A, Reinhard C, Schmidts M, Kispert A, Zariwala MA, Leigh MW, Knowles MR, Zentgraf H, Seithe H, Nurnberg G, Nurnberg P, Reinhardt R, Omran H: **Deletions and point mutations of LRRC50 cause primary ciliary dyskinesia due to dynein arm defects.** *Am J Hum Genet* 2009**, 85**:883-889.

[66] Omran H, Kobayashi D, Olbrich H, Tsukahara T, Loges NT, Hagiwara H, Zhang Q, Leblond G, O'Toole E, Hara C, Mizuno H, Kawano H, Fliegauf M, Yagi T, Koshida S, Miyawaki A, Zentgraf H, Seithe H, Reinhardt R, Watanabe Y, Kamiya R, Mitchell DR, Takeda H: **Ktu/PF13 is required for cytoplasmic pre-assembly of axonemal dyneins.** *Nature* 2008**, 456**:611-616.

[67] Matsuo M, Shimada A, Koshida S, Saga Y, Takeda H: **The establishment of rotational polarity in the airway and ependymal cilia: analysis with a novel cilium motility mutant mouse.** *Am J Physiol Lung Cell Mol Physiol* 2013**, 304**:L736-45.

[68] Mitchison HM, Schmidts M, Loges NT, Freshour J, Dritsoula A, Hirst RA, O'Callaghan C, Blau H, Al Dabbagh M, Olbrich H, Beales PL, Yagi T, Mussaffi H, Chung EM, Omran H, Mitchell DR: **Mutations in axonemal dynein assembly factor DNAAF3 cause primary ciliary dyskinesia.** *Nat Genet* 2012**, 44**:381-9, S1-2.

[69] Tarkar A, Loges NT, Slagle CE, Francis R, Dougherty GW, Tamayo JV, Shook B, Cantino M, Schwartz D, Jahnke C, Olbrich H, Werner C, Raidt J, Pennekamp P, Abouhamed M, Hjeij R, Kohler G, Griese M, Li Y, Lemke K, Klena N, Liu X, Gabriel G, Tobita K, Jaspers M, Morgan LC, Shapiro AJ, Letteboer SJ, Mans DA, Carson JL, Leigh MW, Wolf WE, Chen S, Lucas JS, Onoufriadis A, Plagnol V, Schmidts M, Boldt K, UK10K, Roepman R, Zariwala MA, Lo CW, Mitchison HM, Knowles MR, Burdine RD, Loturco JJ, Omran H: **DYX1C1 is required for axonemal dynein assembly and ciliary motility.** *Nat Genet* 2013**, 45**:995-1003.

[70] Horani A, Druley TE, Zariwala MA, Patel AC, Levinson BT, Van Arendonk LG, Thornton KC, Giacalone JC, Albee AJ, Wilson KS, Turner EH, Nickerson DA, Shendure J, Bayly PV, Leigh MW, Knowles MR, Brody SL, Dutcher SK, Ferkol TW: **Whole-exome capture and sequencing identifies HEATR2 mutation as a cause of primary ciliary dyskinesia.** *Am J Hum Genet* 2012**, 91**:685-693.

[71] Horani A, Ferkol TW, Shoseyov D, Wasserman MG, Oren YS, Kerem B, Amirav I, Cohen-Cymberknoh M, Dutcher SK, Brody SL, Elpeleg O, Kerem E: **LRRC6 mutation causes primary ciliary dyskinesia with dynein arm defects.** *PLoS One* 2013**, 8**:e59436.

[72] Knowles MR, Ostrowski LE, Loges NT, Hurd T, Leigh MW, Huang L, Wolf WE, Carson JL, Hazucha MJ, Yin W, Davis SD, Dell SD, Ferkol TW, Sagel SD, Olivier KN, Jahnke C, Olbrich H, Werner C, Raidt J, Wallmeier J, Pennekamp P, Dougherty GW, Hjeij R, Gee HY, Otto EA, Halbritter J, Chaki M, Diaz KA, Braun DA, Porath JD, Schueler M, Baktai G, Griese M, Turner EH, Lewis AP, Bamshad MJ, Nickerson DA, Hildebrandt F, Shendure J, Omran H, Zariwala MA: **Mutations in SPAG1 cause primary ciliary dyskinesia associated with defective outer and inner dynein arms.** *Am J Hum Genet* 2013**, 93**:711-720.

[73] Zariwala MA, Gee HY, Kurkowiak M, Al-Mutairi DA, Leigh MW, Hurd TW, Hjeij R, Dell SD, Chaki M, Dougherty GW, Adan M, Spear PC, Esteve-Rudd J, Loges NT, Rosenfeld M, Diaz KA, Olbrich H, Wolf WE, Sheridan E, Batten TF, Halbritter J, Porath JD, Kohl S, Lovric S, Hwang DY, Pittman JE, Burns KA, Ferkol TW, Sagel SD, Olivier KN, Morgan LC, Werner C, Raidt J, Pennekamp P, Sun Z, Zhou W, Airik R, Natarajan S, Allen SJ, Amirav I, Wieczorek D, Landwehr K, Nielsen K, Schwerk N, Sertic J, Kohler G, Washburn J, Levy S, Fan S, Koerner-Rettberg C, Amselem S, Williams DS, Mitchell BJ, Drummond IA, Otto EA, Omran H, Knowles MR, Hildebrandt F: **ZMYND10 is mutated in primary ciliary dyskinesia and interacts with LRRC6.** *Am J Hum Genet* 2013**, 93**:336-345.

[74] Merveille AC, Davis EE, Becker-Heck A, Legendre M, Amirav I, Bataille G, Belmont J, Beydon N, Billen F, Clement A, Clercx C, Coste A, Crosbie R, de Blic J, Deleuze S, Duquesnoy P, Escalier D, Escudier E, Fliegauf M, Horvath J, Hill K, Jorissen M, Just J, Kispert A, Lathrop M, Loges NT, Marthin JK, Momozawa Y, Montantin G, Nielsen KG, Olbrich H, Papon JF, Rayet I, Roger G, Schmidts M, Tenreiro H, Towbin JA, Zelenika D, Zentgraf H, Georges M, Lequarre AS, Katsanis N, Omran H, Amselem S: **CCDC39 is required for assembly of inner dynein arms and the dynein regulatory complex and for normal ciliary motility in humans and dogs.** *Nat Genet* 2011**, 43**:72-78.

[75] Becker-Heck A, Zohn IE, Okabe N, Pollock A, Lenhart KB, Sullivan-Brown J, McSheene J, Loges NT, Olbrich H, Haeffner K, Fliegauf M, Horvath J, Reinhardt R, Nielsen KG, Marthin JK, Baktai G, Anderson KV, Geisler R, Niswander L, Omran H, Burdine RD: **The coiled-coil domain containing protein CCDC40 is essential for motile cilia function and left-right axis formation.** *Nat Genet* 2011**, 43**:79-84.

[76] Wirschell M, Olbrich H, Werner C, Tritschler D, Bower R, Sale WS, Loges NT, Pennekamp P, Lindberg S, Stenram U, Carlen B, Horak E, Kohler G, Nurnberg P, Nurnberg G, Porter ME, Omran H: **The nexin-dynein regulatory complex subunit DRC1 is essential for motile cilia function in algae and humans.** *Nat Genet* 2013**, 45**:262-268.

[77] Tokuhiro K, Hirose M, Miyagawa Y, Tsujimura A, Irie S, Isotani A, Okabe M, Toyama Y, Ito C, Toshimori K, Takeda K, Oshio S, Tainaka H, Tsuchida J, Okuyama A, Nishimune Y, Tanaka H: **Meichroacidin containing the membrane occupation and recognition nexus motif is essential for spermatozoa morphogenesis.** *J Biol Chem* 2008**, 283**:19039-19048.

[78] Kott E, Legendre M, Copin B, Papon JF, Dastot-Le Moal F, Montantin G, Duquesnoy P, Piterboth W, Amram D, Bassinet L, Beucher J, Beydon N, Deneuville E, Houdouin V, Journel H, Just J, Nathan N, Tamalet A, Collot N, Jeanson L, Le Gouez M, Vallette B, Vojtek AM, Epaud R, Coste A, Clement A, Housset B, Louis B, Escudier E, Amselem S: **Loss-of-function mutations in RSPH1 cause primary ciliary dyskinesia with central-complex and radial-spoke defects.** *Am J Hum Genet* 2013**, 93**:561-570.

[79] Onoufriadis A, Shoemark A, Schmidts M, Patel M, Jimenez G, Liu H, Thomas B, Dixon M, Hirst RA, Rutman A, Burgoyne T, Williams C, Scully J, Bolard F, Lafitte JJ, Beales PL, Hogg C, Yang P, Chung EM, Emes RD, O'Callaghan C, UK10K, Bouvagnet P, Mitchison HM: **Targeted NGS gene panel identifies mutations in RSPH1 causing primary ciliary dyskinesia and a common mechanism for ciliary central pair agenesis due to radial spoke defects.** *Hum Mol Genet* 2014**, 23**:3362-3374.

[80] Castleman VH, Romio L, Chodhari R, Hirst RA, de Castro SC, Parker KA, Ybot-Gonzalez P, Emes RD, Wilson SW, Wallis C, Johnson CA, Herrera RJ, Rutman A, Dixon M, Shoemark A, Bush A, Hogg C, Gardiner RM, Reish O, Greene ND, O'Callaghan C, Purton S, Chung EM, Mitchison HM: **Mutations in radial spoke head protein genes RSPH9 and RSPH4A cause primary ciliary dyskinesia with central-microtubular-pair abnormalities.** *Am J Hum Genet* 2009**, 84**:197-209.

[81] Davy BE, Robinson ML: **Congenital hydrocephalus in hy3 mice is caused by a frameshift mutation in Hydin, a large novel gene.** *Hum Mol Genet* 2003**, 12**:1163-1170.

[82] Lechtreck KF, Delmotte P, Robinson ML, Sanderson MJ, Witman GB: **Mutations in Hydin impair ciliary motility in mice.** *J Cell Biol* 2008**, 180**:633-643.

[83] Olbrich H, Schmidts M, Werner C, Onoufriadis A, Loges NT, Raidt J, Banki NF, Shoemark A, Burgoyne T, Al Turki S, Hurles ME, UK10K Consortium, Kohler G, Schroeder J, Nurnberg G, Nurnberg P, Chung EM, Reinhardt R, Marthin JK, Nielsen KG, Mitchison HM, Omran H: **Recessive HYDIN mutations cause primary ciliary dyskinesia without randomization of left-right body asymmetry.** *Am J Hum Genet* 2012**, 91**:672-684.

[84] Wallmeier J, Al-Mutairi DA, Chen CT, Loges NT, Pennekamp P, Menchen T, Ma L, Shamseldin HE, Olbrich H, Dougherty GW, Werner C, Alsabah BH, Kohler G, Jaspers M, Boon M, Griese M, Schmitt-Grohe S, Zimmermann T, Koerner-Rettberg C, Horak E, Kintner C, Alkuraya FS, Omran H: **Mutations in CCNO result in congenital mucociliary clearance disorder with reduced generation of multiple motile cilia.** *Nat Genet* 2014**, 46**:646-651.

[85] Boon M, Wallmeier J, Ma L, Loges NT, Jaspers M, Olbrich H, Dougherty GW, Raidt J, Werner C, Amirav I, Hevroni A, Abitbul R, Avital A, Soferman R, Wessels M, O'Callaghan C, Chung EM, Rutman A, Hirst RA, Moya E, Mitchison HM, Van Daele S, De Boeck K, Jorissen M, Kintner C, Cuppens H, Omran H: **MCIDAS mutations result in a mucociliary clearance disorder with reduced generation of multiple motile cilia.** *Nat Commun* 2014**, 5**:4418.

[86] Caspary T, Larkins CE, Anderson KV: **The graded response to Sonic Hedgehog depends on cilia architecture.** *Dev Cell* 2007**, 12**:767-778.
